# Cardiovascular deconditioning and impact of artificial gravity during 60-day head-down bed rest—Insights from 4D flow cardiac MRI

**DOI:** 10.3389/fphys.2022.944587

**Published:** 2022-10-07

**Authors:** Jeremy Rabineau, Margot Issertine, Fabian Hoffmann, Darius Gerlach, Enrico G. Caiani, Benoit Haut, Philippe van de Borne, Jens Tank, Pierre-François Migeotte

**Affiliations:** ^1^ LPHYS, Département de Cardiologie, Université Libre de Bruxelles, Brussels, Belgium; ^2^ TIPs, École Polytechnique de Bruxelles, Université Libre de Bruxelles, Brussels, Belgium; ^3^ Institute of Aerospace Medicine, German Aerospace Center (DLR), Cologne, Germany; ^4^ Electronic, Information and Biomedical Engineering Department, Politecnico di Milano, Milan, Italy

**Keywords:** bed rest, microgravity, artificial gravity, cardiac MRI, 4D flow, cardiovascular deconditioning, wall shear stress, pulse wave velocity

## Abstract

Microgravity has deleterious effects on the cardiovascular system. We evaluated some parameters of blood flow and vascular stiffness during 60 days of simulated microgravity in head-down tilt (HDT) bed rest. We also tested the hypothesis that daily exposure to 30 min of artificial gravity (1 g) would mitigate these adaptations. 24 healthy subjects (8 women) were evenly distributed in three groups: continuous artificial gravity, intermittent artificial gravity, or control. 4D flow cardiac MRI was acquired in horizontal position before (−9 days), during (5, 21, and 56 days), and after (+4 days) the HDT period. The false discovery rate was set at 0.05. The results are presented as median (first quartile; third quartile). No group or group × time differences were observed so the groups were combined. At the end of the HDT phase, we reported a decrease in the stroke volume allocated to the lower body (−30% [−35%; −22%]) and the upper body (−20% [−30%; +11%]), but in different proportions, reflected by an increased share of blood flow towards the upper body. The aortic pulse wave velocity increased (+16% [+9%; +25%]), and so did other markers of arterial stiffness ( CAVI ; ${CAVI}_{0}$ ). In males, the time-averaged wall shear stress decreased (−13% [−17%; −5%]) and the relative residence time increased (+14% [+5%; +21%]), while these changes were not observed among females. Most of these parameters tended to or returned to baseline after 4 days of recovery. The effects of the artificial gravity countermeasure were not visible. We recommend increasing the load factor, the time of exposure, or combining it with physical exercise. The changes in blood flow confirmed the different adaptations occurring in the upper and lower body, with a larger share of blood volume dedicated to the upper body during (simulated) microgravity. The aorta appeared stiffer during the HDT phase, however all the changes remained subclinical and probably the sole consequence of reversible functional changes caused by reduced blood flow. Interestingly, some wall shear stress markers were more stable in females than in males. No permanent cardiovascular adaptations following 60 days of HDT bed rest were observed.

## Introduction

In microgravity as well as simulated microgravity, such as during head-down tilt (HDT) bed rest, a shift of blood volume from the lower to the upper body is observed (Shen and Frishman, 2019). It is then followed by a cascade of adaptations, including a decrease in plasma volume (Blomqvist and Stone, 1983). Together with hypokinesia, in the absence of proper countermeasures, long-term exposure to (simulated) microgravity leads to a decrease in stroke volume (Herault et al., 2000) and cardiac strain (Hoffmann et al., 2021), an increase in resting heart rate ( HR ) (Rabineau et al., 2020), and a post-flight orthostatic intolerance (Lee et al., 2015). On the vascular side, microgravity has also several deleterious effects (Navasiolava et al., 2020). However, current studies remain inconclusive regarding its exact impact on arterial stiffness, an important predictor of cardiovascular risk (Vlachopoulos et al., 2010). While some investigators observed an increased stiffness (Tuday et al., 2007; Hughson et al., 2016; Fayol et al., 2019; Hoffmann et al., 2022), others did not find any significant changes (Palombo et al., 2015; Hoffmann et al., 2019; Möstl et al., 2021).

The countermeasures currently used onboard the International Space Station appear to not optimally counteract the microgravity-induced deconditioning (Hargens et al., 2013; Hurst et al., 2019). Artificial gravity has been suggested as a multi-system countermeasure (Hargens et al., 2013) and is now studied in the context of HDT bed rest (Iwasaki et al., 2001; Iwase, 2005; Stenger et al., 2012). However, only basic investigations have been conducted so far and always for limited periods of exposure to (simulated) microgravity. As a consequence, not only longer studies are needed, but it is also necessary to evaluate the comparative efficacy of different protocols regarding the duration, intensity, and activity performed during exposure to artificial gravity (Hargens et al., 2013).

While assessing the cardiovascular state of an astronaut in space is operationally difficult, ground studies offer a relatively easy access to state-of-the-art technologies. Magnetic resonance imaging (MRI) is a good example of such a technology that can be available during HDT bed rest studies, but not in space. In particular, the recent advances in the field of cardiac MRI allow assessing many new parameters relevant to the cardiovascular health. For instance, 4D flow cardiac MRI can be used to provide a time-resolved 3D velocity field on a volume of interest during free breathing (Stankovic et al., 2014). Among others, this type of protocol enables to accurately measure blood flow rates in the aorta (Houriez--Gombaud-Saintonge et al., 2019). In turn, it allows the estimation of aortic pulse wave velocity ( PWV ) (Markl et al., 2012) and wall shear stress ( WSS ) (Stalder et al., 2008). Both are important markers of the vascular health (Takehara, 2022) and can help better understand the cardiovascular adaptations and potential risks for a subject exposed to a weightless environment.

Accordingly, the general objective of this research was to quantify the cardiovascular deconditioning induced by 60-day exposure to simulated microgravity by HDT bed rest, using 4D flow cardiac MRI. We expected to observe a decrease in the parameters of blood flow, leading to a decrease of aortic WSS and an increase in wall stiffness parameters such as aortic PWV . Additionally, the secondary objective was to evaluate the efficacy of a countermeasure based on artificial gravity, with the hypothesis that it would mitigate the changes in the previously cited parameters of the cardiovascular health, as compared to the control subjects.

## Materials and methods

### Study population and AGBRESA study

The research was part of the study “Artificial Gravity Bed Rest with European Space Agency” (AGBRESA) organized jointly by the American, European, and German space agencies (NASA, ESA, and DLR, respectively). It took place at the: envihab facility of the DLR in Cologne, Germany, between March and December 2019. The AGBRESA study was approved by the Northern Rhine Medical Association (Ärztekammer Nordrhein, application N°2018143) as well as the Federal Office for Radiation Protection (Bundesamt für Strahlenschutz, application N°22464/2018-074-R-G). Written informed consent was obtained from all the subjects prior to starting the protocols.

Twenty-four healthy subjects (8 women) were included after a thorough medical screening (Kramer et al., 2021) (German Clinical Trials Register – DRKS00015677). They were randomly pre-assigned to three groups matched for sex, age, and weight: a continuous artificial gravity group (cAG, *n* = 8, 3 women), an intermittent artificial gravity group (iAG, *n* = 8, 3 women), or a control group (CTRL, *n* = 8, 2 women). Three subjects had to be discharged from the study for medical reasons that were not identified during the screening phase and that were not related to the study protocol. These three subjects have been replaced and the demographics of the final study population can be found in Table 1.

**TABLE 1:** Demographics of the subjects enrolled in the AGBRESA study at baseline (BDC-9). Data are presented as median (first quartile; third quartile), except for the number of subjects (number of females in parentheses). cAG: continuous artificial gravity; iAG: intermittent artificial gravity; CTRL: control.

Group	All	cAG	iAG	CTRL
# subjects (females)	24 (8)	8 (3)	8 (3)	8 (2)
Age (years)	29 (26; 38)	29 (25; 34)	27 (26; 44)	33 (27; 41)
Body mass (kg)	74 (70; 80)	74 (65; 76)	72 (67; 75)	80 (74; 88)
Body height (cm)	176 (167; 182)	175 (165; 176)	175 (163; 184)	179 (173; 183)

Each subject started with a 14-day baseline data collection (BDC) period before being assigned to one of the three aforementioned groups. Then, they began a 60-day strict (i.e., without a pillow under the head) −6° HDT bed rest period, which was followed by a 14-day recovery (R) period. While the subjects were continuously monitored to remain in HDT position during the whole HDT phase, they were authorized to move freely within the ward during the BDC and R phases. Daily water and energy intakes were standardized and controlled throughout the study. The female subjects did not take oral contraceptives and their menstrual cycle was not controlled for. A few subjects experienced some discomfort, especially at the beginning of the HDT phase, including headache, stuffy nose, and back pain. However, these symptoms were never judged as clinically significant, and we believe that they did not impact the results presented thereafter. Additional details regarding the whole protocol are also given in (Kramer et al., 2021).

During the HDT phase, the cAG and iAG groups underwent daily 30-minute exposure to artificial gravity in horizontal (0°) position, using the short-arm human centrifuge available at: envihab. The position of the subject and rotation speed were adapted on an individual basis to achieve a head-to-foot acceleration of 1 g at the center of mass and 2 g at the feet. The cAG group underwent single continuous 30-minute runs on the centrifuge, while, in the iAG group, these 30 min of daily exposure to artificial gravity were distributed in 6 bouts of 5 min separated by 3-minute breaks. The CTRL group was not exposed to artificial gravity during the whole HDT phase. Additional details regarding the artificial gravity protocol, including average spin rate and radii, can be found in (Frett et al., 2020b) and (Kramer et al., 2021).

### 4D flow cardiac MRI

**FIGURE 1:**
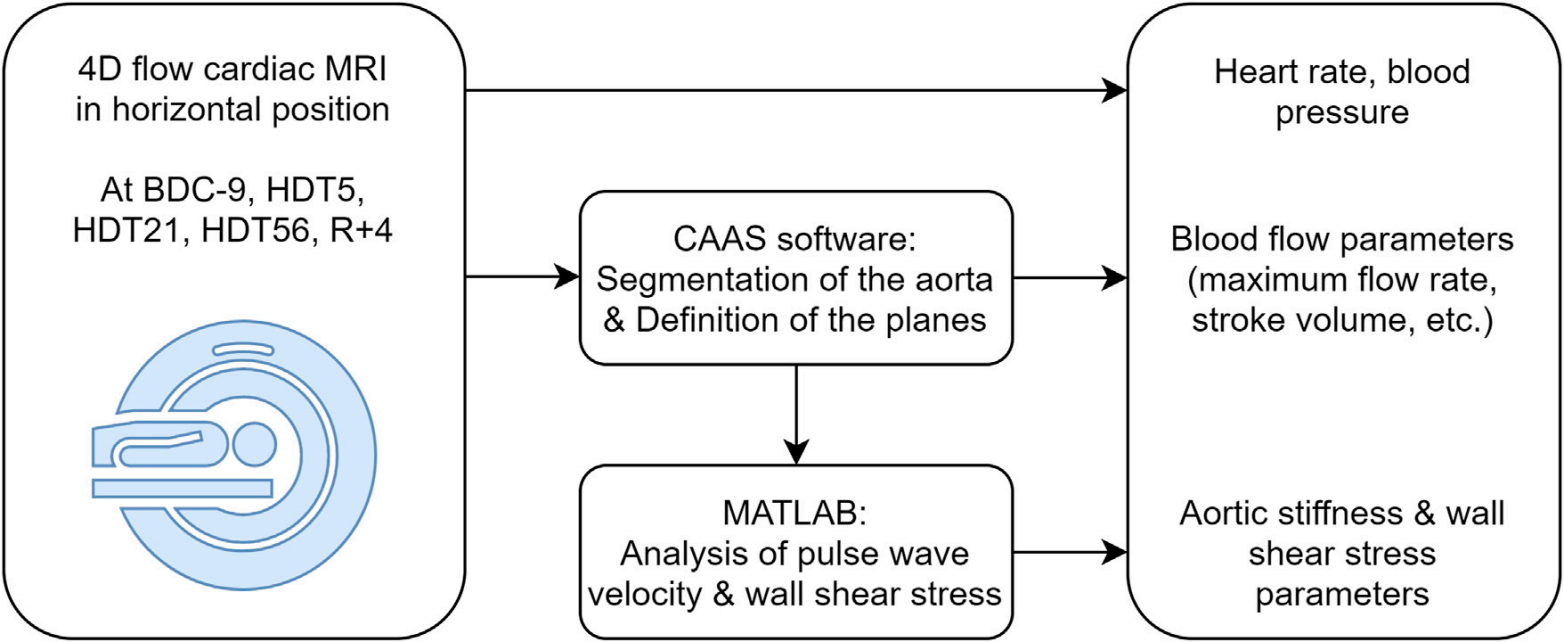
Experimental and analysis procedure. BDC: baseline data collection, HDT: head-down tilt, R: recovery.

The cardiac MRI image acquisition was performed using a Biograph mMR 3-T scanner (Siemens, Erlangen, Germany) with the subject in supine position and no use of contrast agents. The same procedure was repeated at several time points all along the study: before (−9 days, written BDC-9), after (+4 days, written R+4), and during the HDT phase (5, 21, and 56 days, written HDT5, HDT21, and HDT56, respectively). Five subjects had to perform the R+4 data point slightly later (four at +6 days and one at +5 days after the HDT phase) for operational reasons. The cardiac MRI protocol included a free breathing 4D flow acquisition with prospective electrocardiogram- and navigator-gating in a volume encompassing the thoracic aorta. A three-directional encoding velocity of 150 cm/s and the acquisition of 20 images per cardiac cycle were chosen, together with the following parameters: voxel size, 2.375 × 2.375 × 2.00 mm^3^; repetition time, 38.72 ms; echo time, 2.29 ms; flip angle, 7°; acquisition matrix, 90 × 160; and number of slices, 40. In addition, blood pressure at the right brachial artery was measured (Expression MR400, Philips Healthcare, Eindhoven, Netherlands) during the cardiac MRI protocol, a few minutes before the 4D flow acquisition. This protocol is summarized in Figure 1 together with the steps of the analysis.

**FIGURE 2:**
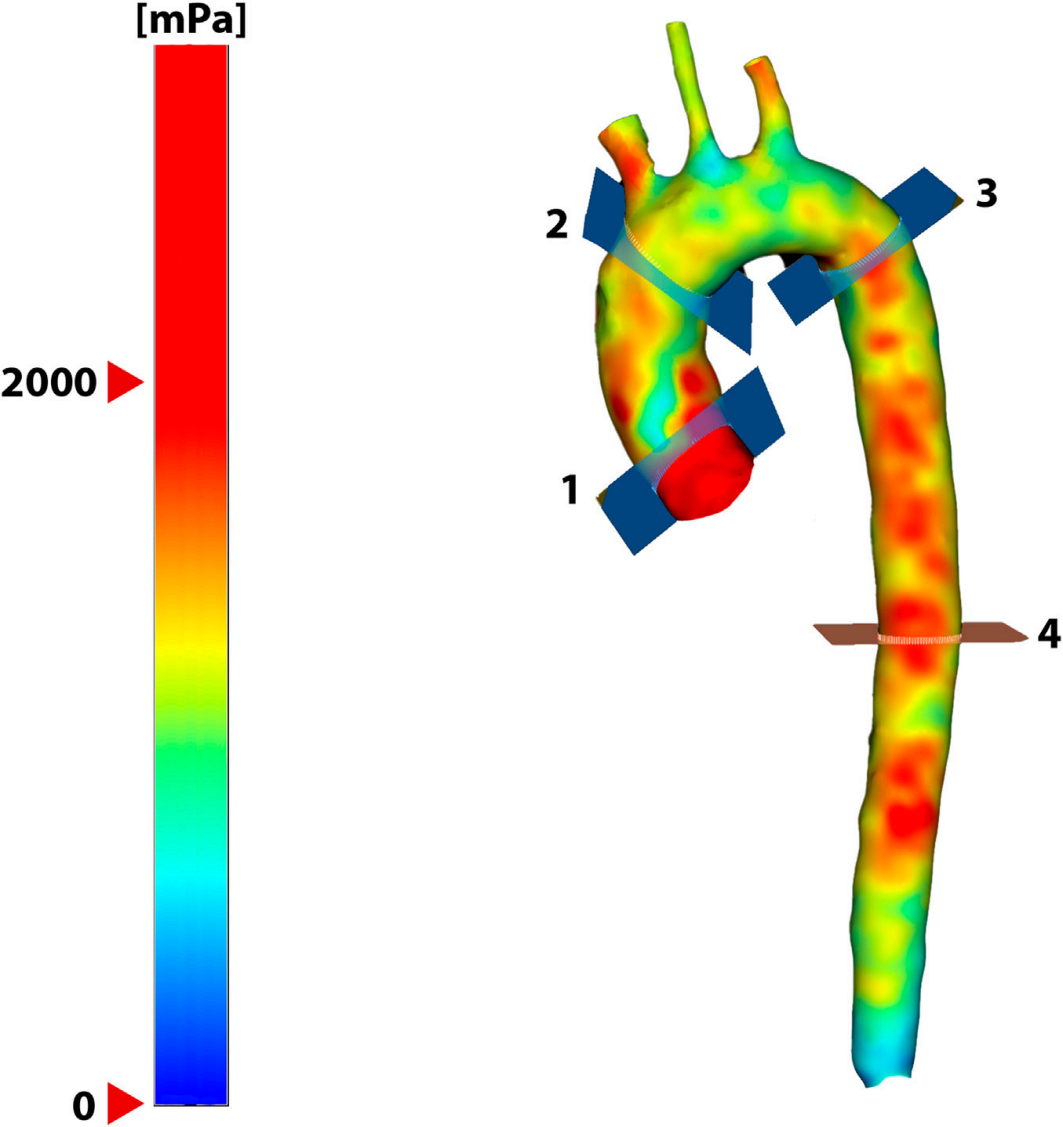
Image reconstruction of the aorta of a representative subject with a map of WSS at a given time of the cardiac cycle. The four reference planes used in the analyses are indicated with their respective numbers.

The post-hoc analysis of the 4D flow images was performed using CAAS MR Solutions 5.2.1 (Pie Medical Imaging BV, Maastricht, The Netherlands) and MATLAB R2018b (The MathWorks, Inc., Natick, Massachusetts, United States). During the pre-processing step, the background phase offsets and velocity aliasing were automatically corrected, as recommended (Dyverfeldt et al., 2015). Then, the aortic volume was semi-automatically segmented and the centerline was extracted using the built-in functionalities available in the commercial software. Four planes were then positioned perpendicularly to the aortic centerline at standardized anatomical landmarks (van Hout et al., 2020) (Figure 2): 1.0 cm after the sinotubular junction (plane 1), just proximal to the brachiocephalic artery (plane 2), 2.0 cm distal to the left subclavian artery (plane 3), and 10.0 cm distal to plane 3 (plane 4).

The total stroke volume ( ${SV}_{tot}$ ) was defined as the total amount of blood passing through the plane 2 in one cardiac cycle, while the total amount of blood passing through the plane 3 in one cardiac cycle was considered as the stroke volume allocated to the lower body ( ${SV}_{low}$ ). The stroke volume allocated to the upper body ( ${SV}_{up}$ ) was then computed as the difference between ${SV}_{tot}$ and ${SV}_{low}$ . The mean HR measured during the 4D flow protocol was used to convert these values in terms of cardiac output ( ${CO}_{tot}$ , ${CO}_{low}$ , and ${CO}_{up}$ , respectively). Based on these values, we then computed the percentage of the cardiac output allocated to the upper body ( ${\%CO}_{up}$ ).

The maximum blood flow rate in the ascending aorta ( $Q_{\max,AA}$ ) was measured on plane 2, while the one in the descending aorta ( $Q_{\max,DA}$ ) was measured on plane 3.

The systemic vascular resistance ( SVR) was calculated as the ratio of the mean arterial pressure to the cardiac output, according to the following formula (Lee et al., 2020):

$$SVR=\frac{SBP+2\;DBP}{3}\frac{1}{{CO}_{tot}}$$ (1)

with SBP and DBP the systolic and diastolic blood pressures, respectively.

The total arterial compliance ( TAC) was calculated as the ratio of ${SV}_{tot}$ to the pulse pressure (Lee et al., 2020):

$$TAC=\frac{{SV}_{tot}}{SBP-DBP}$$ (2)

The aortic pulse wave velocity ( PWV ) was measured between planes 1 and 4. To do so, intermediate planes were defined every 0.6 cm along the centerline, for a total number of 35 ± 2 planes. The associated mean velocity curves were interpolated with a time step of 1 ms using a spline function. A frequency domain method was then used to estimate the pulse transit time ( PTT ) through these planes. To do so, the mean velocity curves were normalized, before their Fourier transforms were computed. Then, the filter transfer function H was derived:

$$H\left(f\right)=\frac{Y\left(f\right)}{X\left(f\right)}$$ (3)

with X(f) and Y(f) the Fourier transforms of the normalized mean velocity curves for the two planes of interest, evaluated at frequency f .

The PTT between these two planes was then given as the sum of the filter group delay weighted by the harmonics of the input signal (Meloni et al., 2014):

$$PTT=\sum_{f}-\frac{d\varphi \left(H\left(f\right)\right)}{df}\frac{{\left|X\left(f\right)\right|}^{2}}{2\pi \;\underset{f}{\sum }{\left|X\left(f\right)\right|}^{2}}$$ (4)

with φ(H(f)) the phase of the filter H(f) .

This procedure was applied to compute PTT between plane 1 and each intermediate plane until plane 4. The values of PTT as a function of the distance with plane 1 populated a scatter plot, on which a linear fit was performed using the least squares method. Finally, the PWV was given by the reciprocal of the slope of the fitted line.

We also computed the cardio-ankle vascular index ( CAVI ) and an updated version of this index ( ${CAVI}_{0}$ ), using the following formulas:

$$CAVI=\frac{2\rho }{SBP-DBP}\ln\left(\frac{SBP}{DBP}\right){PWV}^{2}$$ (5)

with PWV measured in m/s, SBP and DBP measured in Pa, and ρ=1050 kg/m^3^ the volumetric mass density of blood (Pedley, 1980).

$${CAVI}_{0}=2\rho \frac{{PWV}^{2}}{DBP}-\ln\left(\frac{DBP}{P_{0}}\right)$$ (6)

with $P_{0}=13332$ Pa (corresponding to 100 mmHg) a reference pressure equal to the one commonly used in the literature (Giudici et al., 2021).

The intersection between the segmented aorta and each of the four planes previously defined was performed automatically, before extrapolating to the point of zero velocity to properly locate the aortic wall. Assuming a constant blood viscosity of 4.0 mPa.s, the WSS vector was then computed on 90 wall points for each of these planes, as in (Demir et al., 2022). On a given plane, the average of all these 90 WSS amplitudes during the peak systolic phase was defined as ${WSS}_{mean}$ , and the time-averaged WSS over the cardiac cycle as TAWSS . The WSS fluctuations were also evaluated using the oscillatory shear index OSI (Callaghan and Grieve, 2018):

$$OSI=\frac{1}{2}\left(1-\frac{\left\|\int_{0}^{T}WSS\left(t\right)\;dt\right\|}{\int_{0}^{T}\left\|WSS\left(t\right)\right\|\;dt}\right)$$ (7)

with T the average cardiac cycle duration and t the time.

The relative residence time ( RRT ) was computed based on the following equation (Rikhtegar et al., 2012):

$$RRT∼\;\frac{1}{TAWSS\;\left(1-2\;OSI\right)}$$ (8)

where the proportionality constant between RRT and the right-hand side of the expression can be chosen arbitrarily (here it was set to 10^3^).

For the sake of simplicity, in this paper, only the WSS results in the ascending aorta (plane 2) are presented. However, the results in the other planes are similar.

### Statistical analysis

The statistical analysis was performed using GraphPad Prism 9.1.2 (GraphPad Software, San Diego, CA, United States). Three records from two distinct subjects of the CTRL group could not be considered in the analysis due to poor data quality. The effects of time, group, and sex were assessed using a mixed-effects model with the Geisser-Greenhouse correction. If no group and no group × time differences were observed for the metrics studied, the subjects were pooled into a single group of 24 subjects. When the mixed-effects model displayed a significant effect of time, multiple comparisons were performed using the two-stage linear step-up procedure of Benjamini, Krieger, and Yekuteli (Benjamini et al., 2006) to compare each time point to BDC-9. The false discovery rate was set at 0.05. The results are presented as median (first quartile; third quartile), unless otherwise stated. In particular, the relative differences between different conditions are computed based on individual relative differences, before extracting the corresponding quantiles (median and quartiles).

## Results

No group, group × time, group × sex, and group × sex × time differences were found at any time point and for any reported feature. The *p* values corresponding to the group × time effect are given in the penultimate column of Table 2 column of for the blood flow metrics, of Table 3 for the arterial stiffness metrics, and of Table 4 for the WSS metrics. The data from the three groups were then pooled. All the results presented hereafter correspond to the whole set of 24 subjects.

**TABLE 2:** Evolution of blood flow results in the ascending and the descending aorta (AA and DA, respectively) all over the study. Data are presented as median (first quartile; third quartile). The group × time and sex × time effects are displayed in the last columns. HR : heart rate, $Q_{\max}$ : maximum blood flow rate, ${SV}_{tot}$ : total stroke volume, ${SV}_{low}$ : stroke volume allocated to the lower body, ${SV}_{up}$ : stroke volume allocated to the upper body. Their equivalent in terms of cardiac output is noted ${CO}_{tot}$ , ${CO}_{low}$ , and ${CO}_{up}$ , respectively. ${\%CO}_{up}$ : percentage of cardiac output allocated to the upper body. SVR : systemic vascular resistance. BDC: baseline data collection, HDT: head-down tilt, R: recovery. *: Significant difference with BDC-9 using the two-stage linear step-up procedure of Benjamini, Krieger, and Yekuteli with a false discovery rate set at 0.05.

	BDC-9	HDT5	HDT21	HDT56	R+4	group × timeeffect	sex × time effect
HR (bpm)	56 (52; 67)	56 (54; 66)	60 (51; 70)	61 (57; 72) *	65 (57; 73) *	*p* = 0.68	*p* = 0.80
$Q_{\max,AA}$ (ml/s)	444 (356; 512)	387 (351; 473) *	399 (330; 471) *	426 (327; 456) *	412 (374; 491) *	*p* = 0.88	*p* = 0.51
$Q_{\max,DA}$ (ml/s)	311 (256; 341)	265 (222; 308) *	260 (217; 311) *	258 (201; 315) *	294 (235; 322) *	*p* = 0.46	*p* = 0.16
${SV}_{tot}$ (ml)	89 (78; 106)	76 (62; 83) *	73 (57; 84) *	73 (58; 79) *	85 (73; 108)	*p* = 0.85	*p* = 0.22
${SV}_{low}$ (ml)	63 (50; 68)	50 (36; 56) *	44 (36; 52) *	43 (34; 53) *	57 (45; 65) *	*p* = 0.59	*p* = 0.30
${SV}_{up}$ (ml)	27 (25; 38)	25 (22; 31)	27 (22; 32)	27 (20; 32) *	31 (25; 40)	*p* = 0.29	*p* = 0.19
${CO}_{tot}$ (l/min)	5.0 (4.7; 5.6)	4.3 (3.9; 4.7) *	4.1 (3.8; 4.7) *	4.4 (3.8; 4.7) *	5.7 (4.8; 6.2) *	*p* = 0.65	*p* = 0.19
${CO}_{low}$ (l/min)	3.3 (3.2; 3.7)	2.8 (2.4; 3.0) *	2.5 (2.2; 3.1) *	2.7 (2.1; 3.1) *	3.6 (3.0; 4.0)	*p* = 0.28	*p* = 0.30
${CO}_{up}$ (l/min)	1.7 (1.3; 2.2)	1.6 (1.3; 1.8)	1.6 (1.5; 1.7)	1.7 (1.4; 1.9)	2.1 (1.7; 2.4) *	*p* = 0.27	*p* = 0.15
${\%CO}_{up}$ (%)	34 (29; 40)	37 (32; 41) *	38 (34; 41) *	40 (33; 43) *	37 (34; 39) *	*p* = 0.07	*p* = 0.19
SVR (mmHg.min/l)	16.0 (14.8; 17.7)	20.8 (18.4; 23.4) *	20.5 (18.2; 23.8) *	20.3 (18.9; 23.8) *	14.2 (13.1; 16.9) *	*p* = 0.42	*p* = 0.24

**TABLE 3:** Evolution of blood pressure and arterial stiffness results computed between planes 1 and 4 (Figure 2) all over the study. Data are presented as median (first quartile; third quartile). The group × time and sex × time effects are displayed in the last columns. TAC : total arterial compliance, PWV : pulse wave velocity, CAVI : cardio-ankle vascular index, ${CAVI}_{0}$ : updated cardio-ankle vascular index, DBP and SBP : diastolic and systolic blood pressure, respectively, BDC: baseline data collection, HDT: head-down tilt, R: recovery. *: Significant difference with BDC-9 using the two-stage linear step-up procedure of Benjamini, Krieger, and Yekuteli with a false discovery rate set at 0.05.

	BDC-9	HDT5	HDT21	HDT56	R + 4	group × timeeffect	sex × time effect
TAC (ml/mmHg)	1.51 (1.41; 1.87)	1.49 (1.19; 1.62) *	1.41 (1.24; 1.57) *	1.32 (1.08; 1.45) *	1.65 (1.48; 1.84) *	*p* = 0.13	*p* = 0.57
PWV (m/s)	5.3 (4.9; 6.0)	5.9 (5.2; 6.8) *	6.0 (5.2; 7.1) *	6.2 (5.5; 6.9) *	5.2 (4.3; 6.1)	*p* = 0.49	*p* = 0.89
CAVI	5.2 (4.1; 6.2)	5.8 (4.4; 8.2) *	5.8 (4.5; 8.3) *	6.3 (5.0; 9.1) *	5.1 (3.5; 6.5)	*p* = 0.67	*p* = 0.88
${CAVI}_{0}$	7.9 (5.9; 9.2)	8.1 (6.4; 11.4) *	8.1 (6.4; 11.3) *	8.6 (6.6; 12.8) *	6.9 (4.9; 9.5)	*p* = 0.42	*p* = 0.89
DBP (mmHg)	66 (60; 68)	74 (68; 77) *	71 (68; 76) *	70 (68; 75) *	67 (62; 67)	*p* = 0.51	*p* = 0.72
SBP (mmHg)	120 (114; 124)	123 (114; 132) *	126 (115; 131) *	123 (116; 131) *	115 (110; 122) *	*p* = 0.61	*p* = 0.98

**TABLE 4:** Evolution of wall shear stress results computed in the ascending aorta (plane 2, Figure 2) all over the study. Data are presented as median (first quartile; third quartile). The group × time and sex × time effects are displayed in the last columns. ${WSS}_{mean}$ : average amplitude of all the WSS reference points during the peak systolic phase, TAWSS : time-averaged wall shear stress, OSI : oscillatory shear index, RRT : relative residence time, BDC: baseline data collection, HDT: head-down tilt, R: recovery. *: Significant difference with BDC-9 using the two-stage linear step-up procedure of Benjamini, Krieger, and Yekuteli with a false discovery rate set at 0.05.

	BDC-9	HDT5	HDT21	HDT56	R + 4	group × timeeffect	sex × time effect
${WSS}_{mean}$ (mPa)	1331 (1063; 1471)	1165 (1045; 1368) *	1213 (1008; 1293) *	1175 (1005; 1399) *	1299 (1207; 1492)	*p* = 0.73	*p* = 0.46
TAWSS (mPa)	470 (416; 509)	429 (379; 463) *	417 (382; 431) *	405 (386; 437) *	476 (437; 515)	*p* = 0.37	*p* = 0.04
OSI (×10^−2^)	3.28 (2.81; 3.77)	3.21 (2.94; 3.79)	3.24 (2.90; 3.67)	3.24 (2.96; 3.59)	3.21 (2.86; 3.56)	*p* = 0.93	*p* = 0.43
RRT	2.3 (2.1; 2.6)	2.5 (2.3; 2.8) *	2.6 (2.5; 2.8) *	2.6 (2.5; 2.8) *	2.3 (2.1; 2.5)	*p* = 0.57	*p* = 0.04

**FIGURE 3:**
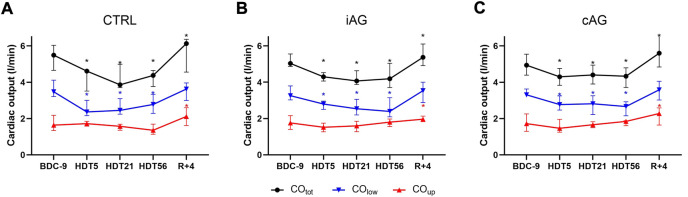
Changes in total cardiac output, as well as cardiac output allocated to the lower and upper body ( ${CO}_{tot}$ , ${CO}_{low}$ , and ${CO}_{up}$ , respectively) in each group all over the study. **(A)** control (CTRL) group; **(B)** intermittent artificial gravity (iAG) group; **(C)** continuous artificial gravity (cAG) group.*: Significant difference with BDC-9 using the two-stage linear step-up procedure of Benjamini, Krieger, and Yekuteli with a false discovery rate set at 0.05. BDC: baseline data collection, HDT: head-down tilt, R: recovery.

The results related to HR , $Q_{\max}$ , SV , CO , and SVR are reported in Table 2. The resting HR was stable during the beginning of the HDT phase and increased only at HDT56 (+8% [+1%; +15%], *p* < 0.001) and at R+4 (+7% [+3%; +15%], *p* < 0.001), when compared to baseline values. In parallel, the peak flow rates in both the ascending and descending aorta decreased at all time points (with −9% [−16%; −2%] in the ascending aorta and –16% [−21%; −5%] in the descending aorta at HDT56 vs. BDC-9, both *p* < 0.001). The values of ${SV}_{tot}$ , ${SV}_{low}$ , and ${SV}_{up}$ decreased at HDT56 vs. BDC-9 (−23% [−33%; −16%], *p* < 0.001; −30% [−35%; −22%], *p* < 0.001; and −20% [−30%; +11%], *p* = 0.03, respectively). For ${SV}_{tot}$ and ${SV}_{low}$ , a smaller (*p* < 0.01) decrease was observed as early as HDT5 (−17% [−23%; −8%] and −21% [−29%; −14%], respectively, both *p* < 0.001). In the case of ${SV}_{low}$ , it even persisted until R+4 (−7% [−14%, +1%], *p* = 0.03). When converting this information in terms of cardiac output, it appears that ${CO}_{up}$ remained unchanged during the whole HDT phase, and even slightly increased at R+4, compared to BDC-9 (+21% [−5%; +40%], *p* = 0.009). In contrast, ${CO}_{tot}$ and ${CO}_{low}$ followed the same evolution as ${SV}_{tot}$ and ${SV}_{low}$ during the HDT phase. These different trends are visible in Figure 3 and induce changes in ${\%CO}_{up}$ , which is higher than baseline during the whole HDT phase (40% [33%; 43%] at HDT56 *versus* 34% [29%; 40%] at BDC-9, *p* = 0.007) and still at R+4 (37% [34%; 39%], *p* = 0.03). SVR was also higher than baseline during the whole HDT phase (+34% [+12%; +52%] at HDT56, *p* < 0.001), but it decreased at R+4 (−7% [−16%; +3%], *p* = 0.006).

The results relevant to blood pressure and arterial stiffness are reported in Table 3. Compared to baseline values, both SBP and DBP increased during the HDT phase (e.g., at HDT56: +2% [+0%; +6%], *p* < 0.001 for SBP , and +11% [+6%; +17%], *p* = 0.01 for DBP ), while TAC decreased (−20% [−12%; −2%] at HDT56, *p* < 0.001). PWV was higher than baseline at all time points of the HDT phase (+16% [+9%; +25%] at HDT56, *p* < 0.001). This was also the case of the two corrected markers based on PWV : CAVI (+23% [+12%; +55%] at HD56, *p* = 0.001) and ${CAVI}_{0}$ (+15% [+4%; +45%] at HDT56, *p* = 0.006). For all these parameters, the changes disappeared quickly during the recovery phase, with even an overshoot at R+4 for SBP (−5% [−6%; −2%], *p* = 0.01) and TAC (+2% [−2%; +14%], *p* = 0.007).

Table 4 summarizes the results relevant to WSS . Both markers of WSS amplitude decreased during the whole HDT phase and came back to baseline values as early as R+4. At HDT56, this decrease reached −5% (−10%; −1%) (*p* = 0.02) for ${WSS}_{mean}$ and −11% (−17%; −4%) (*p* < 0.001) for TAWSS . In contrast, OSI was left relatively unaffected during the whole study (*p* = 0.78). RRT increased during the whole HDT phase (+11% [+4%; +21%] at HDT56 vs. BDC-9, *p* < 0.001) with, again, a quick return to baseline values at R+4.

**FIGURE 4:**
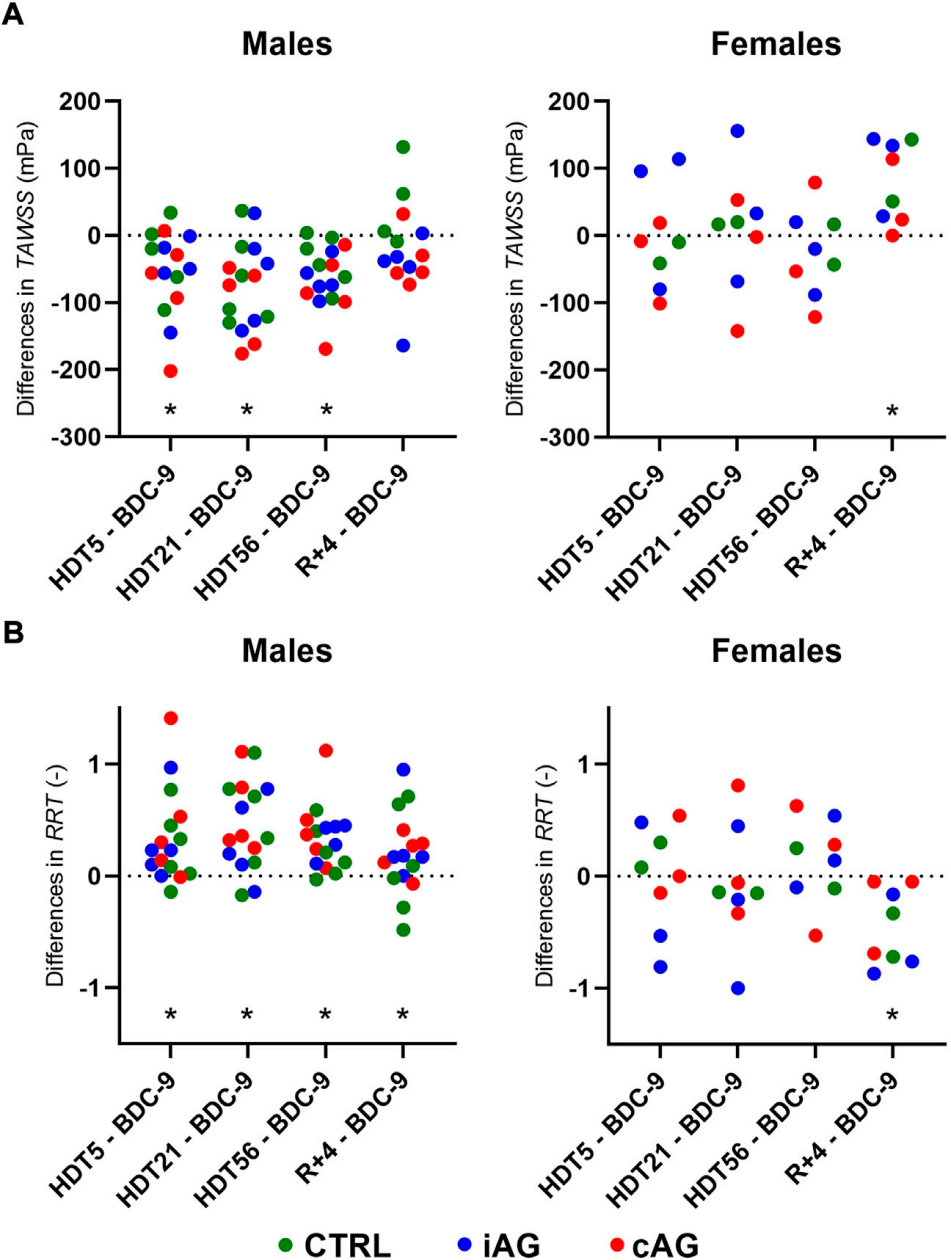
Individual evolution of: **(A)** *TAWSS* and **(B)** *RRT* among males and females and in each group at different timepoints, compared to their baseline values.*: Significant difference with BDC-9 using the two-stage linear step-up procedure of Benjamini, Krieger, and Yekuteli with a false discovery rate set at 0.05. cAG: continuous artificial gravity, iAG: intermittent artificial gravity, CTRL: control, BDC: baseline data collection, HDT: head-down tilt, R: recovery.

The last columns of Table 2, Table 3, and Table 4 present the sex × time effects for the flow, stiffness, and WSS markers, respectively. The only markers presenting a significant sex × time effect are related to WSS : TAWSS and RRT (both *p* = 0.04). The impact of sex on the evolution of TAWSS and RRT during the HDT bed rest study is further depicted in Figure 4, where each timepoint is compared to its baseline value. During the HDT phase, the decrease in TAWSS was observed in males (−13% [−17%; −5%] at HDT56 vs. BDC-9, *p* < 0.001) but not in females. At R+4, TAWSS was back to baseline values in males, while it was increased in females (+13% [+6%; +35%], *p* = 0.007). As for RRT , the increase was observed in males during the HDT phase (+14% [+5%; +21%] at HDT56 vs. BDC-9, *p* < 0.001), while it seemed preserved in females. At R+4, it was still higher than baseline in males, while in females it was decreased (−18% [−27%; −6%], *p* = 0.007).

## Discussion

We used 4D flow cardiac MRI to assess cardiovascular deconditioning among subjects exposed to 60-day strict HDT bed rest. This technique allowed the evaluation of many parameters based on the acquisition of a single volume of interest. The absence of significant differences between the three groups of subjects shows the lack of efficacy of the artificial gravity countermeasure on the estimated parameters. In addition to previously known results relevant to blood flow in (simulated) microgravity, our study confirmed that the cardiac output allocated to the upper and the lower body was differently affected. An increase in several markers of arterial stiffness was observed during the whole HDT phase, with a quick return to baseline values during the recovery phase. The markers related to WSS amplitude decreased during the HDT phase, while RRT increased. All of them were also back to baseline values shortly during the recovery period. Interestingly, the TAWSS and RRT changes were visible only among men during HDT, with opposite changes occurred among women during recovery.

These findings could not only be relevant for astronauts, but also for patients forced to prolonged bedridden immobilization. Indeed, both populations suffer from a multi-system deconditioning that requires to be better understood to find effective countermeasures. In fact, it has been shown that the changes in blood distribution may contribute to the observed bone loss (Colleran et al., 2000), while the arterial adaptations contribute to orthostatic intolerance (Delp, 2007), which can lead to falls and fall-related injuries.

### Interest of 4D flow

4D flow cardiac MRI allows for a very comprehensive assessment of blood flow dynamics in a pre-defined 3D volume throughout the cardiac cycle. Analysis planes can be placed retrospectively at any location within the acquisition volume. Not only does this allow to perform quality checks of the internal consistency of the data (Wentland et al., 2013), but it can also prevent using several standard 2D cine phase contrast protocols, each requiring accurate positioning of the relevant plane while the subject is lying in the scanner. Even though the scanning time of a 4D flow cardiac MRI protocol is longer than the one of a single 2D cine phase contrast acquisition, it can become advantageous in case a series of 2D acquisitions must be scanned (Dyverfeldt et al., 2015). This is especially important in HDT bed rest studies like this one, since it is not desired to ask subjects to remain in horizontal position for too long, especially during the HDT phase, to avoid physiological changes moving from −6° to 0° position. Nevertheless, the measurements are limited to physiological steady state situations.

In addition to the sole blood flow and its traditional metrics, the analysis of the 4D flow images allows the computation of additional parameters, such as the one presented in Table 2 and Table 3. To properly assess PWV , an average of 35 planes all along the aorta were retrospectively defined, which would not have been feasible with 2D cine phase contrast. Other authors have already shown that the evaluation of PWV based on a small number of planes results in less accurate measures (Houriez--Gombaud-Saintonge et al., 2019). Here we used a frequency method because, together with time-frequency methods, they are the most robust to low temporal resolutions (Houriez--Gombaud-Saintonge et al., 2019), correlate well with the traditional carotid-femoral PTT (Bargiotas et al., 2015), and show few outliers (Harloff et al., 2018). Besides this, the distance between each plane can be accurately measured, which is generally not the case for other methods measuring PWV . Finally, 4D flow cardiac MRI image acquisition is performed with the subjects in free breathing, as opposed to traditional acquisitions in breath hold. It is thus more representative of the normal cardiovascular state of the subjects.

### Interest of artificial gravity as a countermeasure

The absence of any visible efficacy of the countermeasure used in this study is not very surprising. Indeed, it is in line with other investigations conducted with the same subjects during the same study, showing its inefficacy: to maintain aerobic exercise capacity (Kramer et al., 2021), to limit the cardiac adaptations (Hoffmann et al., 2021), and to show benefits on the vascular system (Möstl et al., 2021). A 5-day HDT bed rest study with similar artificial gravity parameters (head-to-foot acceleration of 1 g at the center of mass during 30 min per day, continuous or intermittent) led to similar conclusions, with no effect of the countermeasure on plasma volume and aerobic power (Linnarsson et al., 2015), as well as on cardiac function and mass (Caiani et al., 2014). Increasing the daily exposure to 60 min showed some interest regarding orthostatic tolerance, but did not prevent the reduction of plasma volume, stroke volume, and other indices of the cardiac function in another 21-day HDT bed rest study (Stenger et al., 2012).

It has already been reported that this intensity of artificial gravity can be well tolerated by the subjects: in this study, only 10 runs were prematurely stopped due to pre-syncopal symptoms, out of a total of 960 (Frett et al., 2020b). It can be hypothesized that a larger load factor and/or longer exposure time may be used without compromising the safety of the subjects. To this respect, Iwasaki and colleagues have shown that exposure to a daily load factor of 2 g (at the feet) for 1 h could prevent changes in resting HR , sympathovagal balance, arterial cardiac baroreflex gains, and hematocrit caused by 4 days of HDT bed rest (Iwasaki et al., 2001). During another HDT bed rest study of a similar duration, a passive exposure to 1 g (standing position) during 2 h per day partially prevented decreases in orthostatic tolerance, and completely prevented it when the exposure was extended to 4 h per day (Vernikos et al., 1996). Finally, several animal studies have shown that daily exposure to 1 g (obtained by standing) could prevent vascular remodeling (Sun et al., 2004; Gao et al., 2012) as well as myocardial contractility depression (Zhang et al., 2003).

However, artificial gravity alone simply reproduces the effect of standing in a gravity environment, which is not very demanding for the cardiorespiratory system. Thus, this countermeasure is not effective in maintaining aerobic exercise capacity and does not completely protect against the deterioration of muscle function (Kramer et al., 2021). The subjects remain hypokinetic and, in the absence of regular and significant increases in the cardiorespiratory demand (e.g., *via* physical exercises), associated with increases in blood flow and WSS , it is reasonable to expect a similar deconditioning as the one observed in sedentary individuals. Since many countermeasures based on physical exercise have already shown their efficacy to counteract (simulated) microgravity-induced cardiovascular deconditioning (Greaves et al., 2019; Rabineau et al., 2020), another interesting alternative could be to combine artificial gravity and physical exercise (Yang et al., 2010). Indeed, the added value of active *versus* passive exposure to 1 g (controlled walking *versus* standing) regarding the preservation of peak oxygen uptake during a 4-day HDT bed rest study has been demonstrated (Vernikos et al., 1996). In various additional HDT studies from 4 to 14 days, the combination of ergometric exercise with artificial gravity was also effective in preserving plasma volume (Iwase, 2005), stroke volume (Yang et al., 2011), autonomic function (Wang et al., 2011), and orthostatic tolerance (Li et al., 2017). However, even though it has been shown that repetitive jumping exercises were tolerated during short-arm centrifugation (Frett et al., 2020a), involuntary head movements may lead to motion sickness (Young et al., 2001; Iwase, 2005).

An interesting alternative to provide a fluid redistribution towards the lower body, as in a gravity environment, is the use of lower-body negative pressure (LBNP). Indeed, as opposed to the centrifuge, it does not put the subject in a rotating environment. Besides this, it is much smaller and less demanding in terms of power, technical expertise, etc. In addition to increasing LBNP tolerance (Watenpaugh et al., 1994), the combination of LBNP and physical exercise was proven to be more effective than LBNP alone in improving the orthostatic tolerance among ambulatory subjects, especially among females (Evans et al., 2018). During HDT bed rest, combined LBNP and exercise countermeasures have been effective in preserving blood volume (Guinet et al., 2009) and aerobic capacity (Hargens and Richardson, 2009). Watenpaugh and colleagues also showed the interest of adding a short rest period at the end of exercise, while continuing LBNP (Watenpaugh et al., 2007). Indeed, orthostatic stimulation immediately after exercise provides a greater cardiovascular stress than it does prior to exercise. This can be due to many factors including the ongoing skeletal muscle and cutaneous vasodilation, the reduction in vasoconstrictive sympathetic nerve activity, and the cessation of skeletal muscle pumping (Watenpaugh et al., 2007). Still, LBNP does not apply the exact same pressure gradient in the circulation as gravity, including at the level of the cerebral circulation, carotid, and aortic baroreceptors. It is also unable to stimulate the vestibular system, which impacts vestibulo-vascular control mechanisms (Watenpaugh et al., 2007).

### Changes in blood flow

During the HDT phase, the observed increase in HR and decrease in ${SV}_{tot}$ and ${CO}_{tot}$ are in agreement with other investigations conducted during the same study (Kramer et al., 2020; Hoffmann et al., 2021; Möstl et al., 2021). Similar results have also been found in other HDT bed rest studies of various durations (Zhang et al., 1997; Spaak et al., 2005; Caiani et al., 2018; Maggioni et al., 2018; Rabineau et al., 2020). These changes are caused, among others, by the reduced cardiac preload, induced by a reduced volume of circulating blood (Watenpaugh and Hargens, 2011). In addition, the fact that parameters such as ${SV}_{tot}$ continued to decrease between HDT5 and HDT56 could be explained by ventricular remodeling (Perhonen et al., 2001). The same observations were derived from animal models with, among others, hypovolemia, resting tachycardia, and decreased tolerance to lower-body negative pressure (Zhang, 2013). The cardiac mass of rats was not affected by 4 weeks of hindlimb unloading (Ray et al., 2001), whereas such conditions led to a decrease in the systolic function, as expressed by the maximum of dP/dt, possibly due to an impaired Ca^2+^ homeostasis (Cui et al., 2010). 2 weeks of exposure to microgravity has also been shown to lead to a decrease in papillary muscle myofiber area (Goldstein et al., 1992).

The blood supply is not similarly affected in the upper and lower body, as reflected by the increase of ${\%CO}_{up}$ in Table 2, expressing a larger relative decrease in ${SV}_{low}$ than in ${SV}_{up}$ , which is also observed for $Q_{\max,DA}$ *versus* $Q_{\max,AA}$ . These observations seem to suggest that the oxygen supply to the brain is relatively preserved during HDT bed rest, while a potential decrease of ${CO}_{up}$ could also be compensated by the increased hematocrit generally observed in such studies (Linnarsson et al., 2015; Palombo et al., 2015). However, another study has reported a decrease in cerebral blood flow after 26.5 h in −12° HDT position (Kramer et al., 2017), suggesting that the underlying mechanisms may take some time to be fully applied. After 1 month in space, the ratio of carotid and femoral flows has been shown to increase (Herault et al., 2000), while it did not change after 4 days of dry immersion (Greaves et al., 2021), another analogue of microgravity. Previous HDT bed rest studies have already found similar results related to the non-uniform evolution of blood flow parameters in the upper *versus* lower body (Yuan et al., 2015; Caiani et al., 2016; Navasiolava et al., 2020). Similar observations were also made in rats during hindlimb unloading (Colleran et al., 2000). This is an expected result, when the subjects are observed in HDT *versus* horizontal supine position, because of the induced blood shift and its consequences. However, in this study, all the measurements were conducted in horizontal position, suggesting the influence of other parameters. This is also supported by the fact that ${\%CO}_{up}$ was still higher than baseline at R+4.

Even though it was not found in a recent spaceflight study (Lee et al., 2020), the increase of SVR is also a common result of HDT bed rest studies (Zhang et al., 1997; Navasiolava et al., 2020). However, these changes in vascular resistance may be different between the upper and the lower body and thus explain the differences observed in terms of blood flow. Indeed, while an increase in vascular resistance is often observed in the lower limbs (Kamiya et al., 2000; Pawelczyk et al., 2001), it is usually not the case for the upper limbs (Zhang et al., 1997; Yang et al., 2011; Navasiolava et al., 2020). Several authors observed no changes in the main carotid artery blood velocity following exposure to HDT bed rest (Traon et al., 1995; Zhang et al., 1997), while others observed a decreased cerebral blood flow (Sun et al., 2005) that may be caused by an increased cerebral venous pressure (Yang et al., 2011). More generally, many authors reported vascular alterations in the lower body, while vessels of the upper body were left relatively unaffected (Platts et al., 2009; Palombo et al., 2015; Yuan et al., 2015).

### Evolution of arterial stiffness

TAC has been shown to decrease with age, as well as hypertension, diabetes mellitus, and atherosclerosis (Haluska et al., 2008). Here, the observed decrease in TAC during the HDT phase is in agreement with data published by Lee and colleagues on long-duration (>4 months) spaceflights (Lee et al., 2020), even though they did not observe an overshoot following return to the Earth. In another study, Hoffmann and colleagues tested cosmonauts before and after a long-duration flight, and reported a decreased normalized systolic pressure amplification, suggesting an increased compliance of the arterial vasculature post- *versus* pre-flight (Hoffmann et al., 2019). The compliance of the proximal aorta decreased in rats after exposure to 7 days of hindlimb unweighting (Tuday et al., 2007), another analogue of microgravity. A decrease in femoral compliance was also observed after 60 days of HDT bed rest (Yuan et al., 2015), while no significant changes in carotid and brachial artery distensibility were observed after spaceflight (Lee et al., 2020). Interestingly, TAC did not change among the very same subjects as in this study, when it was computed based on an estimation of the aortic pulse pressure and measuring SV in HDT position using ultrasound (instead of MRI in horizontal position, as in this paper) (Möstl et al., 2021). Here, the decrease in TAC is mathematically caused by the decrease in ${SV}_{tot}$ , while the pulse pressure remained relatively stable. Nevertheless, it is not possible to directly conclude that the decrease of TAC is the consequence of an increase in the stiffness of the central arteries. Indeed, by definition, TAC is a global parameter that is a function of central and peripheral arterial stiffness and that can be influenced by many other elements of the circulatory system, including peripheral vasoconstriction.

In contrast, PWV , CAVI , and ${CAVI}_{0}$ represent the local stiffness of the aorta. This is a very interesting element of this study as, in most clinical trials, these three parameters are measured based on the carotid-femoral PWV , thus also including thinner and stiffer vessels (carotid, iliac, and femoral arteries). Other things being equal, a greater PWV indicates stiffer arteries, but there are many confounding factors, including blood pressure (Spronck et al., 2017). Here, we indeed report an increase of DBP and SBP , while other investigations with the same subjects described an increase in the cross-section area of the aorta (ref Mostl et al., 2021). It has been shown that changes of 10 mmHg in the diastolic blood pressure could lead to a difference of 1 m/s in PWV (Spronck et al., 2015). However, this relationship is relatively complex and age-specific (Spronck et al., 2015). To address this interaction issue, Shirai and colleagues introduced the CAVI parameter (Shirai et al., 2006), which, even though named “cardio-ankle”, can be applied to any PWV measurement. An updated version ( ${CAVI}_{0}$ ) has also recently been suggested (Spronck et al., 2017). However, both CAVI and ${CAVI}_{0}$ are highly correlated among healthy subjects (Lin et al., 2020) and it is still not clear which of these two is the best one (Takahashi et al., 2020; Giudici et al., 2021). Still, even using these parameters adjusted for blood pressure, the results in the HDT phase indicate stiffer aortas. The fact that these three markers of aortic stiffness were back to their initial values following only 4 days of recovery, indicates functional rather than structural changes.

The observed changes in these markers of stiffness could be hypothesized to be at least partially due to a decrease in left ventricular ejection time (LVET). Indeed, *in silico* and clinical results have shown that there is an inverse relationship between LVET and PWV (Salvi et al., 2013; Lillie et al., 2015). Besides this, DBP and LVET are independent predictors of PWV (Nürnberger et al., 2003). Published results based on the same study, with the same subjects, have shown a decrease of approximately 30 ms in LVET (Orter et al., 2022), in agreement with other HDT bed rest studies (Caiani et al., 2018; Hoffmann et al., 2022). However, it has been shown that a decrease of ∼40 ms in LVET could lead to an increase of ∼1 m/s in PWV (Nürnberger et al., 2003), which is close to what is observed here. Moreover, LVET has been shown to quickly return to baseline values after HDT bed rest (Caiani et al., 2018; Orter et al., 2022), which is also what is found here regarding PWV .

The median increase in PWV at the end of the HDT phase was 0.9 m/s, which corresponds to about a decade of transient vascular aging (The Reference Values for Arterial Stiffness’ Collaboration, 2010; Harloff et al., 2018). The commonly accepted threshold over which PWV is considered to represent an increased cardiovascular risk is 10 m/s (Williams et al., 2018). While all the subjects were below this limit at BDC-9, one of them exceeded it at HDT21 and HDT56. It may be worth noting that this subject was also the oldest one (54 years old). The median increases in CAVI and ${CAVI}_{0}$ at HDT56 also correspond to more than a decade of healthy vascular aging (Shirai et al., 2019). According to the Japan Society for Vascular Failure, CAVI is considered abnormal when it reaches values greater than 9 (Tanaka et al., 2017). Applying this criterion in this study, 6 subjects had abnormally high values of CAVI at HDT56, even though it was already the case for 2 of them at BDC-9. Overall, these observations agree with recent spaceflight studies suggesting 10–20 years of vascular aging following exposure to microgravity (Arbeille et al., 2016; Hughson et al., 2016).

Hoffmann and colleagues reported no changes in PWV after long-duration spaceflights (Hoffmann et al., 2019), while other teams observed a decreased pulse wave arrival time at the finger on return to Earth, after 1–2 days (Hughson et al., 2016) and after 3–6 days (Baevsky et al., 2007). In addition, it was shown that this pulse arrival time was also decreased compared to baseline during the actual spaceflight (Baevsky et al., 2007). In a study conducted with rats, 7 days of hindlimb unweighting were sufficient to observe a large increase of PWV in the thoracic aorta, but no changes in the abdominal aorta (Tuday et al., 2007). In contrast, no changes were reported in carotid-femoral and carotid to tibial PWV following 4 days of dry immersion in humans (Greaves et al., 2021). Results in other HDT bed rest studies are not clear and, while carotid-femoral PWV was left unchanged following 35 days in HDT position (Palombo et al., 2015), others have found an increase of more than 1 m/s after 60 days of HDT bed rest, with no return to baseline values 1 year after the study (Fayol et al., 2019). A recent study also found a decrease of pulse transit time to the finger following exposure to HDT bed rest (Hoffmann et al., 2022). Interestingly, no changes in brachial-femoral PWV were observed within the same participants as the ones of this paper (Möstl et al., 2021). These different findings might be explained by a different methodology, in particular the position that was chosen during the HDT phase to evaluate the parameters of interest: horizontal here, *versus* HDT in Möstl’s paper. In addition, brachial-femoral PWV includes stiffer vessels, as can be seen by its average value: 9.0 *versus* 5.3 when comparing baseline values. Thus, the evolutions of aortic and brachial-femoral PWV certainly reflect different types of adaptations.

### Changes in wall shear stress

In this study, the observed decrease in the amplitude of aortic WSS is certainly the direct consequence of the decrease in both mean and peak flow in the aorta. Despite this decrease, ${WSS}_{mean}$ and TAWSS remained in the range of normal values for healthy subjects, while OSI was markedly smaller than average in this cohort all along the study (Callaghan and Grieve, 2018; van der Palen et al., 2018; Trenti et al., 2022). In addition to the resting WSS , HDT bed rest studies also prevent regular increases in WSS caused by physical exercise, since lower limbs are underused. Indeed, it has been shown that lower limb exercise caused large increases in TAWSS and large decreases in OSI in the abdominal aorta (Tang et al., 2006).

After 5 weeks of HDT bed rest, Palombo and colleagues did not observe such a decrease in peak wall shear rate in the common carotid and femoral arteries, but they also did not report any changes in mean and systolic flows through these arteries (Palombo et al., 2015). Incidentally, what is reported as WSS in the present paper in fact reflects more the evolution of wall shear rate, since the blood viscosity was considered constant. The literature is not very conclusive regarding the exact evolution of blood viscosity in (simulated) weightlessness and experiments in animal models have found either an increase (Shen et al., 1997; Li et al., 2002; Saunders et al., 2002) or no changes (Ryou, 2003; Hu et al., 2017). Remaining in sitting position during 2 hours has also been shown to increase blood viscosity (Hitosugi et al., 2000). Thus, the reported results may overestimate the actual decrease of ${WSS}_{mean}$ and TAWSS .

When the amplitude of WSS decreases and/or when it becomes more multidirectional, the vascular endothelial cells detect these changes, decrease the release of nitric oxide (NO), and induce the atherogenic processes of the aortic wall (Thijssen et al., 2011a). Computational fluid dynamics model have shown that high WSS magnitude in coronary arteries protected against atherosclerosis (Mahmoudi et al., 2020), while low WSS magnitude led to vascular remodeling and thickening of the vascular wall (Chen et al., 2022). More generally, the magnitude of WSS was found to be inversely correlated with intima-media thickness (Irace et al., 2004).

One could thus argue that the decrease in ${WSS}_{mean}$ and TAWSS is the reason behind the increased carotid and femoral intima-media thickness found in spaceflight (Arbeille et al., 2016). In HDT bed rest, these changes are not systematically reported (Palombo et al., 2015; Yuan et al., 2015), but van Duijnhoven and colleagues noticed them, and observed that a countermeasure based on physical exercise could even reduce them (van Duijnhoven et al., 2010). In rabbits with a high-fat diet meant to cause atherosclerosis, a decrease in the magnitude of WSS was observed already 4 weeks before an increase in intima-media thickness (Zhang et al., 2017). However, in the case of spaceflight, the femoral intima-media thickness was back to baseline values after only 4 days (Arbeille et al., 2016), which seems against the hypothesis of atherosclerotic-based changes. On the contrary, the main reason behind these short-term changes in arterial wall thickness may originate from reversible physiological adaptations, potentially at the level of the smooth muscle cells in the media layer (Thijssen et al., 2011a). This hypothesis is supported by the fact that pharmacological smooth muscle relaxation has been shown to lead to large (10%) and immediate (less than 10 min) reductions in carotid and femoral artery wall thickness (Thijssen et al., 2011b).

An increased vasomotor sympathetic nerve activity and decreased plasma NO has been shown following HDT bed rest (Kamiya et al., 2000). This decreased NO bioavailability increases the vascular tone and promotes the proliferation of smooth muscle cells (Atochin and Huang, 2010). It is thus possible that the observed increase in RRT is the consequence of vascular wall thickening. Indeed, Chen and colleagues have shown the correspondence between the regions of vascular remodeling and those of high RRT (Chen et al., 2017). Other studies also suggested that the areas of high RRT may predict plaque initiation and growth rate (Soulis et al., 2011; Rikhtegar et al., 2012; Hoogendoorn et al., 2020).

In an *ex vivo* experiment, the smooth muscle activation was shown to lead to an increase in aortic stiffness (Franchini et al., 2022). It seems in line with spaceflight findings, where a positive relationship has been observed between carotid intima-media thickness and β-stiffness (Arbeille et al., 2016). This may be the actual reason why an increase in all the markers of aortic stiffness was found during the HDT phase, with such a quick return to baseline values during recovery. However, it has to be expected that the amplitude of changes and the time of recovery may not be the same in conduit vessels like the aorta and in resistance vessels. Still, experiments conducted on hindlimb unloaded rats have shown that the differential regulation of intracellular Ca^2+^ in cerebral and small mesenteric arterial smooth muscle cells was no longer visible after only 3 days of recovery (Xue et al., 2011).

### Differences between male and female subjects

Up to now, female astronauts represent only a small share of the overall population that has flown to space. The same holds true for the participants to studies in the field of altered gravity, including HDT bed rest studies. Only in the recent years, space agencies have tried to reduce this gap by enrolling more female volunteers in such studies, which is even more important as males and females are known to be affected differently by exposure to microgravity (Platts et al., 2014).

In particular, female astronauts are more susceptible to orthostatic intolerance than their male counterparts, with difficulties in maintaining venous return and cardiac output in the upright posture (Harm et al., 2001). Among other factors, this could be due to the indirect vasodilatory effects of estrogen, leading to a smaller vasoconstrictive responsiveness among women (Fritsch-Yelle et al., 1996). In addition, female astronauts experience a larger decrease in plasma volume following spaceflight (Waters et al., 2002), while HDT bed rest studies have come to contradictory findings regarding this parameter (Platts et al., 2014).

Some gender-dependent changes in arterial stiffness may also explain the differences observed in terms of orthostatic tolerance (Tuday et al., 2011). After 6 months in space, it was found that the β-stiffness index in the carotid artery increased more in women than in men, while the opposite was found for the pulse transit time to the finger and to the posterior tibial artery (Hughson et al., 2016). It was also shown, both in HDT bed rest and spaceflight, that arterial stiffness expressed as the inverse of TAC was stable among females, while it increased for males (Tuday et al., 2011). Interestingly, in this study, no gender difference has been found regarding the evolution of TAC . The same holds true for the markers of aortic stiffness such as PWV , CAVI , and ${CAVI}_{0}$ (Table 3). However, the gender-differences found in TAWSS and RRT (Figure 4) seem to indicate a context that is potentially more prone to vascular remodeling (Thijssen et al., 2011a; Chen et al., 2017) in males than in females, for which these parameters are left relatively unchanged.

Cardiac atrophy, as well as the related impact of exercise, has been shown to occur similarly in males and females during HDT bed rest (Dorfman et al., 2007; Platts et al., 2014; Evans et al., 2018). In addition, no gender differences were observed for stroke volume and cardiac output indices following spaceflight (Waters et al., 2002). This is in line with the absence of a significant effect of sex × time observed here on ${SV}_{tot}$ and ${CO}_{tot}$ .

During a 28-day HDT bed rest study, a protocol combining LBNP and exercise was shown to be able to reduce orthostatic tolerance, without reporting differences between men and women (Watenpaugh et al., 2007). In another study with ambulatory subjects, 3 weeks of passive artificial gravity training improved orthostatic tolerance in males but not in females (Stenger et al., 2007). Here, no sex × group and no sex × group × time differences were observed for any of the parameters studied.

Due to the very low number of females included in each group, the relevant statistical power may not have been sufficient to provide a clear conclusion regarding the absence of gender-related differences in the different markers, including the distribution of blood flow and aortic stiffness. Accordingly, we encourage future HDT bed rest studies to enroll more women to be able to answer such questions.

### Limitations

As most of the HDT bed rest studies, this research is limited by the number of included subjects. In particular, the distribution in three different groups of 8 subjects may have hidden a potential small effect of the applied countermeasure. While, it has been recommended to evaluate gender differences related to artificial gravity countermeasures (Evans et al., 2018), here there were only 2 to 3 women in each group. Even if no gender × group (results not presented) and only a few gender × time differences were observed, it is not possible to come with a clear conclusion regarding gender differences, given the very low number of female subjects in each group.

In such studies involving many experiments and paired groups, it is very difficult to control the timing of a specific protocol with regard to the menstrual cycle phase. Even HDT bed rest studies including only women often lack this type of control (Guinet et al., 2009). However, differences in the balance of ovarian hormones during the menstrual cycle have an impact on autonomic functions (Dimitriev et al., 2007), which can impact orthostatic tolerance. In addition, the menstrual cycle has been reported to have an impact on resting heart rate (Moran et al., 2000), on systolic and diastolic functions of both ventricles (Zengin et al., 2007), as well as on smooth muscle sensitivity to NO and whole-body arterial compliance (Williams et al., 2001), while central arterial stiffness remains relatively preserved (Ounis-Skali et al., 2006). We encourage including more women in future HDT bed rest studies so that the exact impact of this confounding factor can be better understood.

The 4D flow cardiac MRI acquisitions were all started after approximately 40 min in horizontal supine position, even during the HDT phase. On the one hand, this may not be representative of the actual cardiovascular state of the subjects at each step of the study, but on the other hand, it also helps to better compare the results at the different time points.

HDT bed rest results cannot readily be extended to actual microgravity as some dissimilarities remain. Indeed, the body is not in absolute weightlessness during HDT bed rest studies, which may have an impact on some cardiovascular parameters. For instance, Lee and colleagues argued that unweighting of the neck tissue overlying the common carotid artery may play a key role in space, allowing the vessel to expand, while gravity still acts upon the neck tissue in HDT bed rest and the diameter of the common carotid artery remains unchanged (Lee et al., 2020).

In addition, the fitness status of the participants was lower than the one of the astronaut population, especially when correcting for the age (Moore et al., 2014; Kramer et al., 2021). Indeed, at the time of their first flight, astronauts are usually older by about one decade than the participants of this study (Kovacs and Shadden, 2017). Thus, precautions should be taken when trying to extend these results to the whole astronaut population.

Several parameters were computed based on the brachial blood pressure, while the aortic blood pressure would have been more relevant, if it had been possible to measure it. This is particularly the case of CAVI and ${CAVI}_{0}$ .

Finally, the 4D flow cardiac MRI protocol had a relatively low temporal resolution, especially for low heart rates, with only 20 images per cardiac cycle. It has been shown that this could lead to an underestimation of peak flow rate (Dyverfeldt et al., 2015), but also of OSI (Cibis et al., 2016). However, this does not challenge our conclusions regarding blood flow and WSS , as we discussed relative differences. In addition, the chosen method for the evaluation of aortic PWV has been proven to lead to only negligible differences between an acquisition at 50 images per cardiac cycle, and a subset of this acquisition corresponding to 20 images per cardiac cycle (Houriez--Gombaud-Saintonge et al., 2019).

We believe that these limitations did not preclude any of the conclusion of this research.

## Conclusion

We demonstrated the potential of 4D flow cardiac MRI to assess the longitudinal evolution of several cardiovascular parameters through a 60-day strict HDT bed rest study. To our knowledge, this is the first time that 4D flow cardiac MRI is used in this context, thus overcoming limitations of other imaging techniques, and enabling the evaluation of many aortic parameters that were never measured in simulated microgravity. In the absence of significant differences between the different groups studied, we can conclude that the effects of the applied artificial gravity countermeasures were either absent or too weak to reach statistical significance. Future evaluations of potential countermeasures based on artificial gravity should either increase the load factor, the time of exposure, or combine it with physical exercise. The observed changes in blood flow confirmed the different adaptations occurring in the upper and lower body during exposure to (simulated) microgravity. A larger share of blood volume was dedicated to the upper body during the HDT phase, which helps cerebral perfusion. All the markers of arterial stiffness indicated a more rigid aorta during the HDT phase, which may be caused by the decrease in the magnitude of WSS and/or by other hemodynamic changes including a decrease in LVET. Interestingly, some changes in WSS parameters were observed only in males during the HDT phase, while females experienced opposite changes during recovery. No other gender-related differences were observed but future studies should investigate these aspects in more detail by including more female subjects. All the changes in aortic stiffness and WSS parameters remained subclinical, as probably the sole consequence of functional—rather than structural—changes. Indeed, most of the modifications tended to or returned to baseline already after 4 days of recovery, thus indicating no permanent cardiovascular adaptations following 60 days of strict HDT bed rest. Still, these observations trigger the need to evaluate these parameters or their proxies during long duration space travels, including potential remote monitoring, to verify if changes remain subclinical or if they translate in clinical manifestation, thus posing the need to assess a potential risk evolution profile.

## Data Availability

The raw data supporting the conclusion of this article will be made available by the authors, without undue reservation.

## Glossary

**AA**: Ascending aorta
**AGBRESA**: Artificial gravity bed rest with European space agency
**BDC**: Baseline data collectionc
**AG**: Continuous artificial gravity group
**cAG**: Continuous artificial gravity group
CAVI: Cardio-ankle vascular index
${CAVI}_{0}$: Updated cardio-ankle vascular index
**CTRL**: Control group
${CO}_{low}$: Cardiac output allocated to the lower body
${CO}_{tot}$: Total cardiac output
${CO}_{up}$: Cardiac output allocated to the upper body
**DA**: Descending aorta
DBP: Diastolic blood pressure
**DLR**: Deutsches Zentrum für Luft- und Raumfahrt (German aerospace center)
**ESA**: European space agency
g: Earth’s gravitational acceleration
**HDT**: Head-down tilt
HR: Heart rate
**iAG**: Intermittent artificial gravity group
**LVET**: Left ventricular ejection time
**MRI**: Magnetic resonance imaging
**n**: Number of subjects
**NASA**: National aeronautics and space administration
**NO**: Nitric oxide
OSI: Oscillatory shear index
PTT: Pulse transit time
PWV: Pulse wave velocity
$Q_{\max,AA}$: Maximum blood flow rate in the ascending aorta
$Q_{\max,DA}$: Maximum blood flow rate in the descending aorta
**R**: Recovery
RRT: Relative residence time
SBP: Systolic blood pressure
${SV}_{low}$: Stroke volume allocated to the lower body
${SV}_{tot}$: Total stroke volume
${SV}_{up}$: Stroke volume allocated to the upper body
SVR: Systemic vascular resistance
t: Time
T: Duration of a cardiac cycle
TAWSS: Time-averaged wall shear stress
WSS: Wall shear stress
${WSS}_{mean}$: Average amplitude of all the WSS reference points during the peak systolic phase
${\%CO}_{up}$: Percentage of cardiac output allocated to the upper body
ρ: Volumetric mass density of blood
